# Impact of CT-determined low kidney volume on renal function decline: a propensity score-matched analysis

**DOI:** 10.1186/s13244-024-01671-2

**Published:** 2024-04-05

**Authors:** Tomohiro Kikuchi, Shouhei Hanaoka, Takahiro Nakao, Yukihiro Nomura, Harushi Mori, Takeharu Yoshikawa

**Affiliations:** 1 Department of Computational Diagnostic Radiology and Preventive Medicine, The University of Tokyo Hospital, 7-3-1 Hongo, Bunkyo-Ku, Tokyo, 113-8655 Japan; 2 Department of Radiology, Jichi Medical University School of Medicine, 3311-1 Yakushiji, Shimotsuke, Tochigi, 329-0498 Japan; 3 Department of Radiology, University of Tokyo Hospital, 7-3-1 Hongo, Bunkyo-Ku, Tokyo, Japan; 4 Center for Frontier Medical Engineering, Chiba University, 1-33 Yayoicho, Inage-Ku, Chiba, Japan

**Keywords:** Chronic renal insufficiency, Glomerular filtration rate, Kidney, X-ray computed tomography

## Abstract

**Objectives:**

To investigate the relationship between low kidney volume and subsequent estimated glomerular filtration rate (eGFR) decline in eGFR category G2 (60–89 mL/min/1.73 m^2^) population.

**Methods:**

In this retrospective study, we evaluated 5531 individuals with eGFR category G2 who underwent medical checkups at our institution between November 2006 and October 2017. Exclusion criteria were absent for follow-up visit, missing data, prior renal surgery, current renal disease under treatment, large renal masses, and horseshoe kidney. We developed a 3D U-net-based automated system for renal volumetry on CT images. Participants were grouped by sex-specific kidney volume deviations set at mean minus one standard deviation. After 1:1 propensity score matching, we obtained 397 pairs of individuals in the low kidney volume (LKV) and control groups. The primary endpoint was progression of eGFR categories within 5 years, assessed using Cox regression analysis.

**Results:**

This study included 3220 individuals (mean age, 60.0 ± 9.7 years; men, *n* = 2209). The kidney volume was 404.6 ± 67.1 and 376.8 ± 68.0 cm^3^ in men and women, respectively. The low kidney volume (LKV) cutoff was 337.5 and 308.8 cm^3^ for men and women, respectively. LKV was a significant risk factor for the endpoint with an adjusted hazard ratio of 1.64 (95% confidence interval: 1.09–2.45; *p* = 0.02).

**Conclusion:**

Low kidney volume may adversely affect subsequent eGFR maintenance; hence, the use of imaging metrics may help predict eGFR decline.

**Critical relevance statement:**

Low kidney volume is a significant predictor of reduced kidney function over time; thus, kidney volume measurements could aid in early identification of individuals at risk for declining kidney health.

**Key points:**

• This study explores how kidney volume affects subsequent kidney function maintenance.

• Low kidney volume was associated with estimated glomerular filtration rate decreases.

• Low kidney volume is a prognostic indicator of estimated glomerular filtration rate decline.

**Graphical Abstract:**

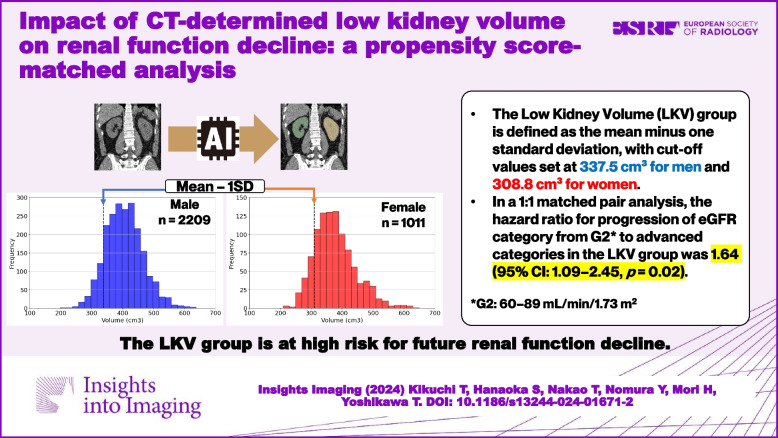

**Supplementary Information:**

The online version contains supplementary material available at 10.1186/s13244-024-01671-2.

## Introduction

Renal function naturally diminishes with age, especially after the age of 20 to 40 years; however, the rate of decline varies among individuals [1]. According to the Kidney Disease Improving Global Outcomes (KDIGO) guidelines for chronic kidney disease (CKD), individuals can be classified based on their estimated glomerular filtration rate (eGFR) as follows: G1 (> 90 mL/min/1.73 m^2^), G2 (60–89 mL/min/1.73 m^2^), G3a (45–59 mL/min/1.73 m^2^), G3b (30–44 mL/min/1.73 m^2^), G4 (15–29 mL/min/1.73 m^2^), and G5 (< 15 mL/min/1.73 m^2^) [2]. Those with eGFR category G3a or higher have an increased risk of complications such as cardiovascular diseases and mortality [3]. Managing these risks often involves adherence to certain guidelines including dietary restrictions [4]. Consequently, preserving renal function for those with eGFR category G2 is vital for their quality of life; furthermore, identifying and managing individuals in the broader population at the highest risk of renal decline is of utmost importance.

Several factors such as sociodemographic, genetic, and metabolic dynamics have been associated with eGFR decline [5]. However, while several studies have established a mild-to-moderate correlation between kidney volume and eGFR [6–8], it remains unclear whether kidney volume can offer valuable prognostic information. In the context of renal transplantation, low kidney volume has been reported to negatively impact post-transplant eGFR for both the donors and recipients [9, 10]. Given this established relationship, we hypothesized that a relatively low kidney volume could be associated with subsequent eGFR decline in populations without known kidney diseases. If kidney volume is identified as a predictive factor for CKD progression, it could help target the high-risk groups that should receive more intensive interventions to maintain kidney function.

Therefore, this study aimed to investigate the effect of renal volume ascertained at the time of initial examination on the subsequent progression to G3a and beyond in individuals with eGFR at G2. To this end, we retrospectively analyzed data derived from a longitudinal medical checkup program and employed a propensity score matching approach.

## Methods

This retrospective study was approved by the Ethics Review Board of our institution. This study complied with the Declaration of Helsinki and was structured according to the Strengthening the Reporting of Observational Studies in Epidemiology (STROBE) guidelines [11]. Written informed consent was obtained from all participants.

### Participants

Our database included information on demographics, health history, blood test results, urine test results, and whole-body CT images of healthy individuals who participated in our medical checkup program. All tests on individuals were performed on the same day in principle. Note that whole-body CT scans are an optional component for individuals who are 40 and above, conducted solely for those who opt for this examination and have provided informed consent, and are not conducted for research purposes. Using our database, we implemented a specific selection process for the study cohort. The inclusion criteria were as follows: (i) individuals with an initial consultation date between November 2006 and October 2017; (ii) those who underwent whole-body CT; and (iii) those with eGFR category G2. The exclusion criteria were as follows: (i) individuals who were absent for follow-up visits; (ii) those with missing data for the analysis items listed in Table 1; (iii) those with a history of renal surgery; (iv) those with current renal disease under treatment including hydronephrosis, nephritis, pyelonephritis, and acute kidney injury; and (v) those with CT findings of a renal mass of > 4 cm or horseshoe kidney.

**Table 1:** Baseline participant characteristics in unmatched and propensity score matched groups

	Before matching			After matching		
	LKV group *n* = 408	Control *n* = 2812	*p*	LKV group *n* = 397	Control *n* = 397	*p*
Sex (male)	270 (66.2%)	1939 (69.0%)	0.28	266 (67.0%)	255 (64.2%)	0.46
Age (years old)	55.8 ± 10.4	56.0 ± 9.9	0.77	55.7 ± 10.3	55.7 ± 10.3	0.93
Body height (cm)	163.8 ± 8.4	165.7 ± 8.6	< *0.01*	164.1 ± 8.2	163.6 ± 8.3	0.38
Body weight (kg)	61.7 ± 13.4	67.0 ± 12.6	< *0.01*	62.2 ± 13.2	61.9 ± 13.0	0.78
BMI	22.8 ± 3.7	24.3 ± 3.4	< *0.01*	22.9 ± 3.7	22.9 ± 3.5	1.00
Smoking						
Never	211 (51.7%)	1188 (42.2%)	< *0.01*	203 (51.1%)	199 (50.1%)	0.25
Past	125 (30.6%)	967 (34.4%)		123 (31.0%)	141 (35.5%)	
Current	72 (17.6%)	657 (23.4%)		71 (17.9%)	57 (14.4%)	
Antihypertensive medication	62 (15.2%)	582 (20.7%)	*0.01*	61 (15.4%)	64 (16.1%)	0.85
Systolic blood pressure (mmHg)	122 ± 16.6	122.6 ± 16.1	0.59	122.1 ± 16.6	122.6 ± 16.4	0.66
Antidiabetic medication	12 (2.9%)	173 (6.2%)	*0.01*	12 (3.0%)	11 (2.8%)	1.00
HbA1c (%)	5.6 ± 0.6	5.8 ± 0.7	< *0.01*	5.6 ± 0.6	5.6 ± 0.5	0.69
EF (%)	65.6 ± 6.6	65.9 ± 6.0	0.53	65.6 ± 6.6	65.5 ± 5.8	0.79
eGFR (mL/min/1.73 m^2^)	72.9 ± 7.8	76.1 ± 7.7	< *0.01*	73.1 ± 7.7	73.3 ± 7.5	0.74
Serum albumin (g/dL)	4.2 ± 0.2	4.1 ± 0.2	0.36	4.2 ± 0.2	4.2 ± 0.3	0.82
Serum uric acid (mg/dL)	5.8 ± 1.4	5.8 ± 1.4	0.53	5.8 ± 1.3	5.7 ± 1.4	0.18
Hematuria (qualitative)						
−	384 (94.1%)	2664 (94.7%)	0.19	374 (94.2%)	361 (90.9%)	1.00
1 +	9 (2.2%)	78 (2.8%)		9 (2.3%)	17 (4.3%)	
2 +	8 (2.0%)	45 (1.6%)		7 (1.8%)	11 (2.8%)	
3 +	6 (1.5%)	19 (0.7%)		6 (1.5%)	5 (1.3%)	
4 +	1 (0.2%)	7 (0.2%)		1 (0.3%)	3 (0.8%)	
Proteinuria (qualitative)						
−	395 (96.8%)	2717 (96.6%)	1.00	385 (97.0%)	385 (97.0%)	1.00
1 +	8 (2.0%)	58 (2.1%)		8 (2.0%)	9 (2.3%)	
2 +	3 (0.7%)	25 (0.9%)		2 (0.5%)	2 (0.5%)	
3 +	1 (0.2%)	11 (0.4%)		1 (0.3%)	1 (0.3%)	
4 +	1 (0.2%)	1 (< 0.1%)		1 (0.3%)	0 (0%)	

*LKV* Low kidney volume, *BMI* Body mass index, *HbA1c* Hemoglobin A1c, *EF* Ejection fraction, *eGFR* Estimated glomerular filtration rate

*p* < 0.05 is indicated in italics for significance

### Data collection and endpoint

For the selected participants, the following first-visit data were extracted: demographics and health history, including medication information, blood tests, urine tests, and CT data. After the initial visit, each time a participant attended a checkup program, both eGFR and elapsed days since the first visit were documented. The primary study endpoint was “progression to the advanced eGFR categories (detection of eGFR below 60).” Data were tracked and collected for up to 5 years from the initial consultation. Cases with no confirmed checkups after 5 years were considered censored as of the date of the last visit. The CT images in our database were acquired with the participants’ arms down, using the following parameters: tube voltage, 120 kV; field of view, 500 mm; matrix size, 512 × 512; and voxel size, 0.98 × 0.98 × 1.25 mm.

### Kidney CT volumetry and grouping

Using a 3D U-net model [12], we previously developed an automated system for kidney volume extraction from unenhanced CT images. Comprehensive details of the network’s training methods and results are presented in Additional file 1: Appendix 1. Participants in the study cohort were categorized into the low kidney volume (LKV) group or the control group based on the results of CT volumetry of the kidney, using the “mean minus one standard deviation (SD)” as cutoff. Recognizing the documented variations in kidney volume between sexes [13–15], we determined the cutoff values separately for each sex. Note that this cutoff value was determined using the results of a preliminary examination conducted on the 3220 individuals in our study cohort (Additional file 1: Appendix 2).

### Propensity score matching

To address potential confounders and minimize selection bias, we employed a propensity score (PS) matching analysis. These scores were derived using binary logistic regression to determine the likelihood of participants being assigned to either the LKV or the control group. According to previous literature [5, 16] that highlighted the association of certain variables with renal volume or identified variables as established risk factors for CKD progression, we chose the following independent variables from the initial visit data, as listed in Table 1: sex, age, height, weight, body mass index (BMI), smoking status, antihypertensive medication, systolic blood pressure (SBP), antidiabetic medication, hemoglobin A1c (HbA1c), ejection fraction (EF), eGFR, serum albumin, serum urine acid, hematuria, and proteinuria. Nearest-neighbor (Greedy-type) 1:1 matching without replacement was performed using a caliper width of 0.2 SD of the propensity score.

### Statistical analysis

Detailed participant demographics are presented for both the entire cohort and the propensity-matched participants. Continuous variables were compared using the Student’s *t*-test, while categorical variables were compared using the chi-square test. Within the PS-matched cohort, cumulative incidence curves (1 minus Kaplan–Meier survival curve) for endpoint occurrence were plotted for each group and compared using the log-rank test. Additionally, a Cox regression analysis was conducted. The following variable cutoff values and categorization methods were primarily adopted from previous studies [5, 16] or general classification criteria: sex, age (categorized by every 10 years), BMI (cutoffs at 18.5 and 25), smoking (never/past or current), hypertension (SBP > 130 mm/Hg and/or on medication), diabetes (HbA1c > 6.5 and/or on medication), heart failure (EF < 50%), low serum albumin level (< 4.1 g/dL), high serum uric acid level (> 6 mg/dL), hematuria (2 + or greater), and proteinuria (2 + or greater). All of the variables in the univariate analyses were entered into a multivariate Cox regression analysis to assess their significance as independent predictors. Hazard ratios (HR) and respective 95% confidence intervals (CI) were compared. The above statistical analyses were performed using JMP Pro 17.0.0 software (JMP Statistical Discovery LLC, Cary, NC, USA). Statistical significance was set at *p* < 0.05.

## Results

**Fig. 1:**
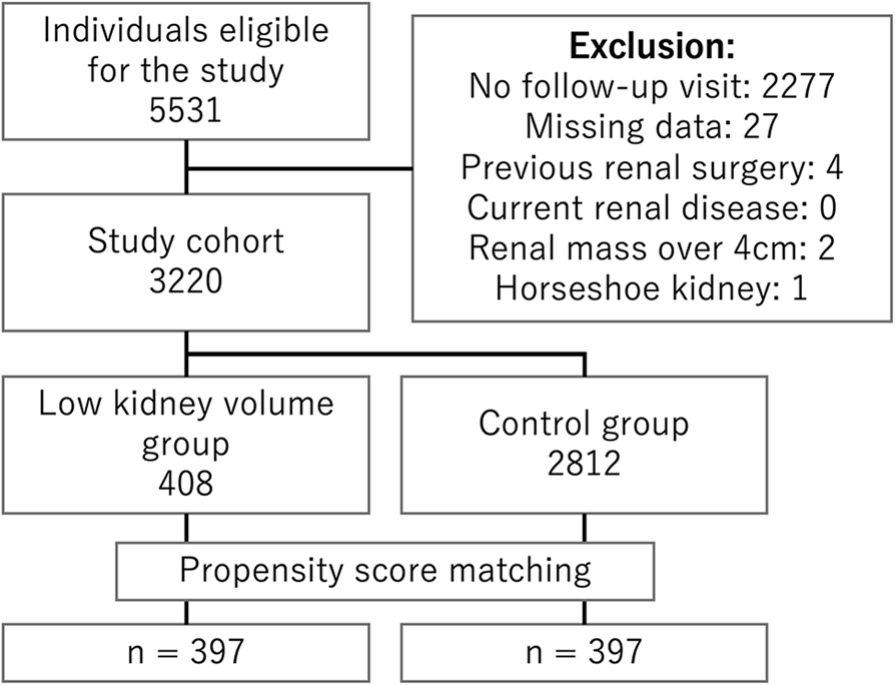
Flowchart of participant selection process

**Fig. 2:**
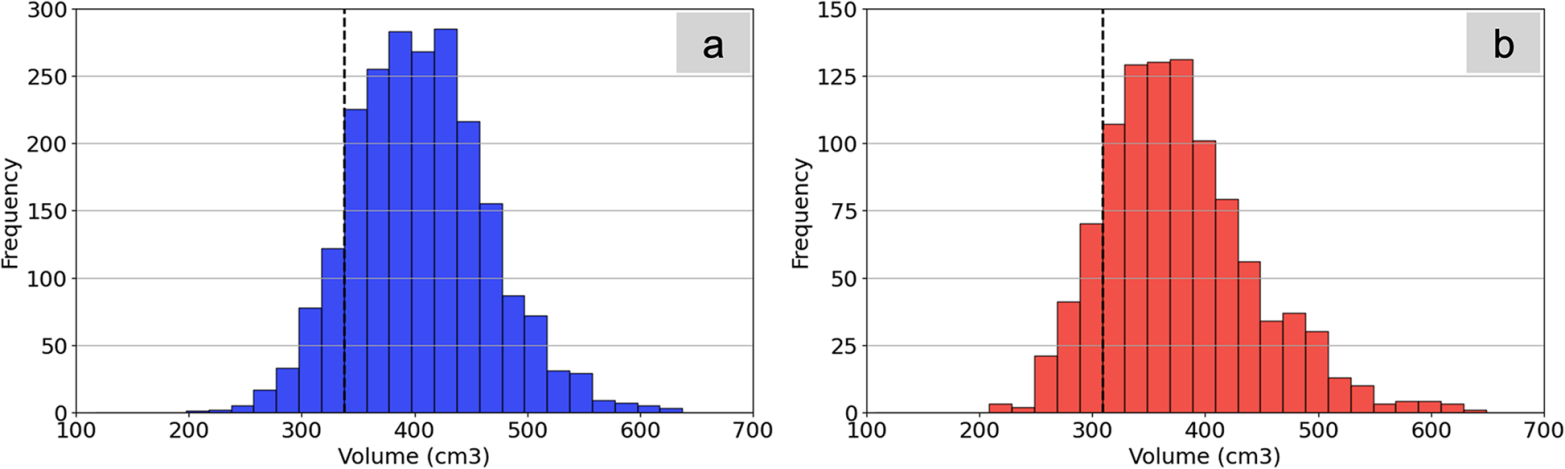
Results of kidney volumetry of the 3220 study participants. On the left (**a**) indicated in blue, is the histogram for men (404.6 ± 67.1 cm^3^). On the right (**b**) indicated in red, is the histogram for women (376.8 ± 68.0 cm^3^). Within each histogram, the vertical dashed line represents the cutoff value calculated as the mean minus one standard deviation.

In total, 5531 individuals were eligible for the study; after applying the exclusion criteria, 3220 participants were selected, with a mean age of 60.0 ± 9.7 years (2209 men). Figure 1 presents the flowchart of the participant selection process. In this study cohort, the kidney volume was 404.6 ± 67.1 cm^3^ in men and 376.8 ± 68.0 cm^3^ in women. A histogram of kidney volumetry for the study cohort is shown in Fig. 2; the cutoff value was calculated as the mean minus 1 SD (The LKV cutoff was 337.5 cm^3^ for men and 308.8 cm^3^ for women). Based on the results of CT volumetry of the kidneys, 408 participants were categorized as having a low kidney volume, while 2812 were classified as controls. After 1:1 propensity score matching, 397 participants from each group were included in the final analysis. Table 1 presents the distribution of each variable before and after propensity score matching, as well as the *p*-values comparing the LKV and control groups. After PS matching, no significant differences were observed between the two groups in the distribution of the variables used for matching.

**Fig. 3:**
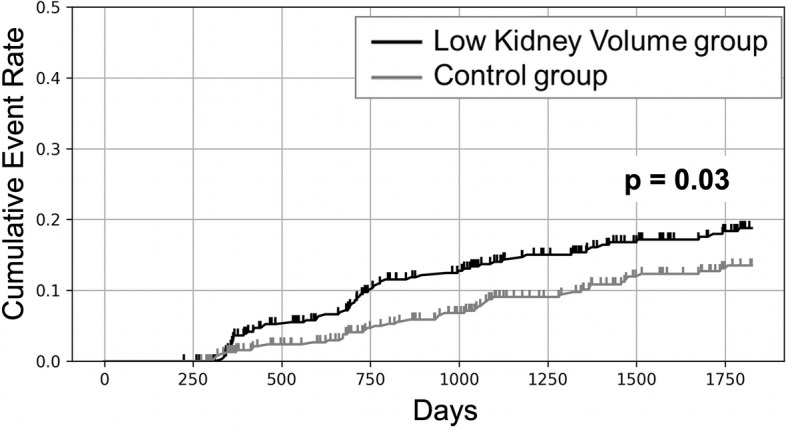
Cumulative hazard curves for endpoints. The cumulative incidence curve was defined as 1, Kaplan–Meier survival curve. Small vertical marks indicate censoring

Of the 794 PS-matched individuals (397 in each group), with a total of 3015.5 person-years of follow-up (average 3.8 years, maximum 5.0 years), 106 individuals experienced progression of eGFR categories. Figure 3 shows the cumulative incidence curves. The curve of the LKV group was positioned higher than that of the control group. The log-rank test revealed a statistically significant difference (*p* = 0.03). Table 2 summarizes the outcomes of the Cox regression analysis. In the univariate analysis, the HR of the LKV group associated with low kidney volume for progression of eGFR categories was 1.52 (95% CI: 1.03–2.23, *p* = 0.04). This association persisted in the multivariate analysis, where the LKV group demonstrated an HR of 1.64 (95% CI: 1.09–2.45, *p* = 0.02). The group with eGFR ranging from 60–69 demonstrated a strong association with the progression of eGFR categories to G3 with HRs of 65.13 (95% CI: 9.08–467.24, *p* < 0.01) in the univariate analysis and 56.27 (95% CI:7.81–405.35, *p* < 0.01) in the multivariate analysis. The age group of ≥ 70 years and high serum uric acid group showed a significant increase in HR, demonstrating HRs of 3.12 (95% CI: 1.70–5.71, *p* < 0.01) and 1.70 (95% CI: 1.16–2.50, *p* = 0.01) respectively, in the univariate analysis but did not show significance in the multivariate analysis.

**Table 2:** Cox regression analysis for a progression of eGFR categories within 5 years among propensity score-matched participants

	Univariate			Multivariate		
	HR	95% CI	*p*	HR†	95% CI	*p*
Low kidney volume group	1.52	1.03–2.23	*0.04*	1.64	1.09–2.45	*0.02*
Sex (male)	1.12	0.74–1.69	0.59	1.00	0.61–1.63	0.99
Age (ref: 40–49 years old)						
50–59	1.56	0.92–2.64	0.10	1.35	0.77–2.37	0.30
60–69	1.72	0.98–3.03	0.06	1.42	0.75–2.70	0.28
≧ 70	3.12	1.70–5.71	< *0.01*	1.91	0.93–3.92	0.08
BMI (ref: 18.5–25)						
> 25	1.14	0.72–1.80	0.58	1.13	0.68–1.86	0.64
< 18.5	0.83	0.33–2.04	0.68	0.80	0.31–2.09	0.65
Current smoker	0.67	0.37–1.22	0.19	0.72	0.38–1.36	0.31
Hypertension	1.36	0.93–1.99	0.12	1.27	0.83–1.94	0.27
Diabetes	0.71	0.26–1.94	0.51	0.45	0.15–1.32	0.14
Low EF	1.45	0.20–10.38	0.71	1.71	0.22–13.49	0.61
eGFR (ref: ≧ 80 mL/min/1.73 m^2^)						
70–79	5.47	0.70–42.71	0.11	4.94	0.63–38.64	0.13
60–69	65.13	9.08–467.24	< *0.01*	56.27	7.81–405.35	< *0.01*
Low serum albumin	1.39	0.93–2.07	0.11	1.08	0.70–1.67	0.73
High serum uric acid	1.70	1.16–2.50	*0.01*	1.37	0.87–2.15	0.18
Hematuria (≧ 2 +)	1.18	0.48–2.91	0.71	1.78	0.66–4.81	0.26
Proteinuria (≧ 2 +)	1.02	0.14–7.32	0.98	0.60	0.07–4.85	0.63

*HR* Hazard ratio, *BMI* Body mass index, *CI* Confidence interval, *EF* Ejection fraction, *eGFR* estimated glomerular filtration rate

*p* < 0.05 is indicated in italics for significance

## Discussion

The objective of this study was to investigate the influence of kidney volume measured at the initial visit on subsequent eGFR decline in individuals with mild eGFR reduction (G2 category). We found that the adjusted HR associated with low kidney volume for a progression of eGFR categories was 1.64 (95% CI: 1.09–2.45, *p* = 0.02), indicating that low kidney volume was a significant risk factor even after adjusting for eGFR values per se and other known risk factors.

eGFR is known to decrease for various reasons, and early interventions for high-risk groups with declining kidney function may contribute to improved patient outcomes by preventing disease progression [1, 5, 17, 18]. While previous studies have identified various factors influencing CKD progression such as sociodemographic, behavioral, genetic, cardiovascular, and metabolic factors [5, 16, 19], our findings add a new dimension to this established list by suggesting the potential of imaging factors such as kidney volume, as prognostic indicators for eGFR decline. Several studies have investigated factors associated with kidney volume in the general population. Kidney volume has been weakly to moderately correlated with factors such as sex, height, weight, BMI, and age [7, 13, 14], and there is a potential association between low birth weight and this measure in adulthood [20]. However, the relationship between kidney volume and longitudinal health outcomes remains poorly understood. Furthermore, while marked atrophy of the kidneys in end-stage CKD is well-known, little attention has been paid to the imaging evaluation of the intermediate conditions leading up to it. This highlights a gap in research on the progression of kidney volume changes and their impact on health over time. Our central research question was whether a relatively low kidney volume is a mere manifestation of functional decline or an indication of future renal outcomes. To address this question, our study employed PS matching to ensure a balanced comparison of risk factors for eGFR decline between the LKV and control groups. After matching, neither group showed significant differences in variables, indicating controlled variations in baseline characteristics. The LKV group had a consistently higher cumulative incidence curve than its matched control group, and this difference was statistically significant according to the log-rank test (*p* = 0.03). Upon conducting a multivariate Cox regression analysis, it was evident that being in the LKV group (HR: 1.64, 95%CI: 1.09–2.45) and having an eGFR value ranging 60–69 (HR: 56.27, 95%CI: 7.81–405.35) were significant risk factors for migration to G3. Following this, as a direction for future research, we believe that investigating the effects of early and intensive interventions or follow-ups for such high-risk groups for CKD progression could lead to the accumulation of insights that are beneficial for the preservation of renal function.

The significance of knowing the volume of areas of interest in medical imaging has been demonstrated in various contexts: volume of kidneys as highlighted in this study; relationship between Alzheimer’s disease and hippocampus volume [21]; correlation between liver cirrhosis and liver volume [22]; and detailed evaluations of tumors [23, 24] and aneurysms [25, 26]. Volumetric analysis offers a more precise three-dimensional assessment than traditional linear measurements [27]. Conventionally, the analysis has been labor-intensive, requiring manual delineation of a certain workstation/software for each individual case, making large-scale analysis and evaluation challenging. However, deep-learning-based segmentation is now streamlining this process [28, 29]. By training or utilizing appropriate deep-learning models, we can efficiently and swiftly extract areas of interest from medical images, circumventing the cost and time of manual methods. Our study is the largest to date to use CT volumetry of the kidney for analytical research [7, 10, 13, 14, 20, 30, 31]. In addition to volumetry, the extraction of regions of interest from medical images yields diverse quantitative data such as CT density in CT scans, signal intensity in MRI, standardized uptake value (SUV) in PET, and even radiomic features [32]. This technology facilitates large-scale image analysis and is likely to be increasingly integrated with epidemiological research, aiding in the generation of high-quality evidence.

This study has several limitations. First, it was based on a single ethnic group from one institution. These participants underwent fee-based checkups, possibly indicating a health-conscious cohort. Therefore, the generalizability of these results is limited. Second, a comprehensive verification of the segmentation results for all the cases was not conducted. In particular, CT scans were performed with the arms down, and streak artifacts may have affected the quality of renal imaging and segmentation results. However, given the similarity in kidney volumetry results with those of prior studies [7, 31, 33, 34] and their near-normal distribution, we posit that the overall impact of segmentation errors is not significant. As examples, CT images of kidney slices of three randomly selected cases used in the analysis (cases segmented by the deep-learning model) and their segmentation results are attached in Additional file 1: Appendix 3. Third, our study overlooked the impact of small lesions. Those with a maximum diameter of up to 4 cm were included; if assumed to be spherical, this represents an ignored volume increase of up to 33.5 mL. It cannot be definitively stated that this has no impact on the results. Furthermore, as a future direction, if a system capable of segmenting both lesions and normal renal tissue were simultaneously developed allowing for the measurement of “volume of normal part of the kidney,” this could potentially yield more detailed and novel insights. Fourth, certain background factors may not have been adjusted for, and the presence of unmeasured confounding factors that strongly influence kidney volume and eGFR cannot be ruled out.

In conclusion, low kidney volume defined as below the sex-specific mean minus 1 standard deviation emerged as a significant predictor of a progression of eGFR categories within 5 years, with an adjusted hazard ratio of 1.64 (95% CI: 1.09–2.45, *p* = 0.02). This underscores the prognostic value of imaging in the early detection of CKD risk. Further research is warranted to explore whether targeted interventions in individuals with low kidney volume can arrest CKD progression.

### Supplementary Information

**Supplementary Material 1.**

## Data Availability

The data presented in this study are available on request from the corresponding author. The data are not publicly available due to informed consent not presupposing the individual data's public availability.

## Abbreviations

BMI: Body mass index
CI: Confidence intervals
CKD: Chronic kidney disease
EF: Ejection fraction
eGFR: Estimated glomerular filtration rate
HbA1c: Hemoglobin A1c
HR: Hazard ratios
LKV: Low kidney volume
SBP: Systolic blood pressure
SD: Standard deviation
