# Supplementary material for: Impact of CT-determined low kidney volume on renal function decline: a propensity score-matched analysis
Source: Insights Imaging. 2024 Apr 5;15:102. doi: 10.1186/s13244-024-01671-2 (PMC10997556; doi:10.1186/s13244-024-01671-2)

Impact of CT-Determined Low Kidney Volume on Renal Function Decline: A Propensity Score-Matched Analysis
ELECTRONIC SUPPLEMENTARY MATERIAL

Appendix 1A: Deep Neural Network Model and Hyperparameter Details

We defined our model using the PyTorch version 1.13.0 (https://pytorch.org/docs/1.13/) and Monai version 1.2.0 (https://docs.monai.io/en/1.2.0/). The model and hyperparameters are specified as follows.

```
model = monai.networks.nets.UNet( spatial_dims=3,
                                  in_channels=1,
                                  out_channels=3,
                                  channels=(16, 32, 64, 128, 256),
                                  strides=(2, 2, 2, 2),
                                  num_res_units=2,
                                  norm=Norm.BATCH
                                  )
loss_function = DiceLoss(to_onehot_y=True, softmax=True)
optimizer = torch.optim.Adam(model.parameters(), 1e-4)
```

During training, input images were cropped to a size of (128,128,128) using "monai.transforms.RandCropByPosNegLabel". For data augmentation, we employed the "monai.transforms.RandAffine" transformation.

Appendix 1B: Model Training and Results

Training

We utilized 107 cases for training, which were different from those included in the analysis in this study and split in a 6:2:2 ratio for the training, validation, and test cohorts. Ground truth labels for the kidneys were created by a radiologist with 7 years of experience using 3D Slicer software (version 5.1.0, https://www.slicer.org). The training was terminated when the loss function on the validation set showed no improvement over 30 epochs. The best model on the validation set prior to this point was considered the final trained model.

Results

The Dice coefficient of the test set was 0.948 ± 0.023. When the kidney volume was calculated using ground truth labels, the result was 382.8±48.7 cm^3. By contrast, using the predicted labels the volume was 383.1±42.3 cm^3. The paired t-test yielded a *p*-value of 0.9206, indicating no statistically significant difference between the two measurements.

Appendix 2: Preliminary study to determine cutoff values for kidney volume

To date, no study has clearly defined a cutoff value for renal volume. Therefore, a preliminary study was conducted to determine a provisional cutoff value. For the 3220 participants in our study cohort, we calculated the deviation from the mean kidney volume for each sex (expressed in terms of ±SD) and set cut-offs at -2.0, -1.5, -1, -0.5, ±0, +0.5, +1.0, +1.5, +2.0 SD. We then generated the receiver operating characteristic curve for these cut-offs against the outcome of progression to a higher category of CKD (which is the primary endpoint of this study) (Figure S1). Upon examining the Youden index to determine the cut-off that maximized it, we found -1SD to be the point (followed by -0.5SD, ±0) and chose -1SD as our cut-off.

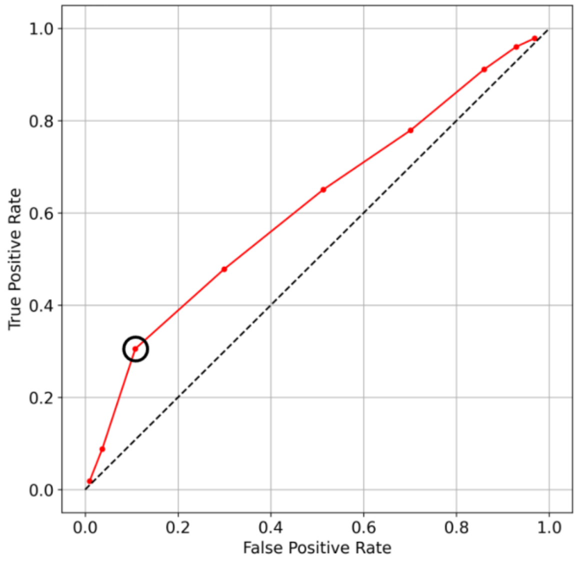

Figure S1: The receiver operating characteristic curve for the association between kidney volume (deviation from the mean) and the outcome

The point highlighted with a black circle is the point of -1SD, where the Youden index is the maximum.

Appendix 3: Three examples of CT images at the kidney level with and without their segmentation results (Figure S2)

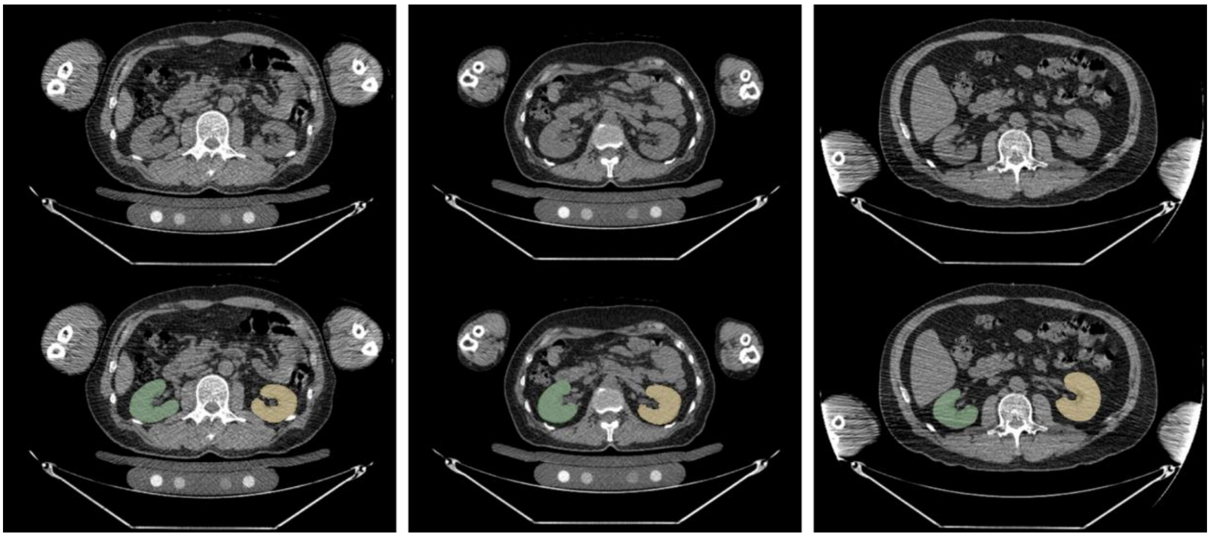

Supplement: Supplementary file 1 — Supplementary Material 1. [file 13244_2024_1671_MOESM1_ESM.pdf]
